# Case Series of Perforated Keratomycosis after Laser-Assisted In Situ Keratomileusis

**DOI:** 10.1155/2020/7237903

**Published:** 2020-09-15

**Authors:** Taher Eleiwa, Eyup Ozcan, Samar Abdelrahman, Omar Solyman, Abdelrahman M. Elhusseiny, Gehad Youssef, Ahmed Bayoumy

**Affiliations:** ^1^Bascom Palmer Eye Institute, Miller School of Medicine, University of Miami, Miami, FL, USA; ^2^Department of Ophthalmology, Faculty of Medicine, Benha University, Egypt; ^3^Net Eye Medical Center, Gaziantep, Turkey; ^4^Department of Clinical Pathology, Faculty of Medicine, Benha University, Egypt; ^5^Department of Ophthalmology, Texas Childrens Hospital, Baylor College of Medicine, Houston, TX, USA; ^6^Department of Ophthalmology, Kasr Al-Ainy Hospitals, Cairo University, Cairo, Egypt

## Abstract

**Background:**

Fungal keratitis is an extremely rare complication of laser vision correction resulting in poor visual outcomes. Amniotic membrane transplantation should be kept in mind in eyes with corneal perforation prior to penetrating keratoplasty.

**Aim:**

To assess the outcomes of multilayered fresh amniotic membrane transplantation (MLF-AMT) in patients with severe keratomycosis after laser-assisted in situ keratomileusis (LASIK). *Study design*. Hospital-based prospective interventional case series.

**Methods:**

Five eyes of 5 patients were included in the study. All cases underwent microbiological scrapings from residual bed and intrastromal injections of amphotericin (50 mcg/mL), with flap amputation if needed, followed by topical 5% natamycin and 0.15% amphotericin. MLF-AMT was performed after corneal perforation. Later, penetrating keratoplasty (PK) was performed when corneal opacity compromised visual acuity. The outcome measures were complete resolution of infection, corneal graft survival, and best-corrected visual acuity (BCVA).

**Results:**

The mean age of patients was 22 ± 1.2 years with 4/5 (80%) were females. The mean interval between LASIK and symptom onset was 8.8 ± 1 day, and the mean interval between symptom onset and referral was 14 ± 1.4 days. Potassium hydroxide (KOH) smears showed filamentous fungi, and Sabouraud's medium grew Aspergillus in all cases. Melted flaps were amputated in 4 (80%) cases. MLF-AMT was performed in all cases due to corneal perforation after a mean time of 12.4 ± 1.2 days of antifungals. In all cases, complete resolution of infection was seen 26 ± 1.8 days after MLF-AMT, and optical PK was done at a mean of 2.4 months later. No postoperative complications after MLF-AMT or PK were observed, with a 0% incidence of corneal graft rejection, and a final BCVA ranged from 20/20 to 20/80 after a mean follow-up of 14 ± 1.1 months.

**Conclusion:**

MLF-AMT is a safe and valid option to manage corneal perforation during keratmycosis treatment to avoid emergency therapeutic keratoplasty.

## 1. Introduction

Laser-assisted in situ keratomileusis (LASIK) has become the leading laser vision correction method in the last two decades. Besides its high accuracy and predictability, infectious keratitis after LASIK has been reported as a rare and devastating complication [1–3]. In the survey of American Cataract and Refractive Surgery (ASCRS), the prevalence of microbial keratitis was reported as a rate of 0.034% in more than 300,000 procedures, and 10% of those cultured microorganisms were fungal agents [4]. Fungal keratitis is a vision-threatening condition that may cause corneal melting and perforation [5, 6]. Although variable applications of antifungals, such as fluconazole, natamycin, amphotericin B, and voriconazole have been implemented, their efficacy is limited because of the fungistatic nature of most of topical antifungals with poor penetration to the deeper corneal layers, and the development of drug resistance [6]. Therefore, therapeutic keratoplasty (TPK) is usually required to protect the integrity of the globe and improve visual acuity [7]. However, recurrence of fungal infections and low graft survival rates after TPK are still challenging [8–10].

In this prospective interventional case series, we evaluated the procedure of multilayered fresh amniotic membrane transplantation (MLF-AMT) prior to penetrating keratoplasty (PK) in patients with severe fungal keratitis after LASIK. The outcome measures were complete resolution of infection, corneal graft survival, and best-corrected visual acuity.

## 2. Case Report

### 2.1. Methods

Our study included all patients with refractory microbiologically diagnosed keratomycosis after LASIK who presented to our outpatient clinic at Benha University hospital, from January 2017 to July 2018. Cases were referred to our hospital after doing LASIK surgery elsewhere. This study was approved by the Institutional Review Board of Benha University hospital, and informed consents were taken from all patients in accordance with the Declaration of Helsinki. Patients having coincident viral or bacterial keratitis, recent eye trauma, recent contact lens use, and previous ocular pathology with any systemic illness were excluded from the study.

At admission, a full history was taken, including age, gender, and time intervals after LASIK surgery till the start of complaint, referral to our department. Clinical data recorded included best-corrected visual acuity (BCVA), clinical features of corneal infiltrate, ocular tension, anterior chamber (AC) inflammatory activity, and corneal perforation characteristics.

At the first visit, under topical anesthesia, a flap lift was done; then, collection of specimens from underneath the flap, the residual stromal bed, AC hypopyon, and amputated parts of macerated flaps was done and sent for microbiological analysis. Processing of all samples was performed according to a standardized protocol in an operating room under complete aseptic measures [11]. 10% potassium hydroxide (KOH) wet mount was reported immediately, and once fungal etiology was verified, amphotericin (50 mcg/mL) interface wash, with intrastromal injection, was performed in the same sitting. Then, according to the antifungal sensitivity tests, topical 5% natamycin and amphotericin 0.15% with oral itraconazole were given in all patients. MLF-AMT was indicated for corneal perforation and AC collapse.

Fresh human amniotic membrane (AM) was acquired from women who were seronegative for viral hepatitis, human immunodeficiency virus, and syphilis before undergoing elective caesarean section. Membranes were manually peeled from the underlying placental tissue and washed in 0.9% normal saline 4 times, then rinsed once in 0.025% sodium hypochlorite. After trimming the amnion along with the underlying chorion into pieces (5 × 5 cm^2^ in size), it was stored in normal saline containing penicillin (50,000 U) and streptomycin (1 gm/400 cc of saline) at 4°C not surpassing a period of 48 h. During the surgery, the amnion was bluntly separated from the chorion and rinsed in gentamicin containing normal saline before usage.

Under peribulbar anesthesia, debridement of the necrotic tissue at the ulcer base was done, and samples were sent for microbial analysis. The AM was adjusted to fit the ulcer dimensions and put one layer superimposed on another layer afterwards, all with stromal face down. Using interrupted 10/0 nylon sutures, all layers were secured in place. The AC was reformed, and the hypopyon was aspirated and sent for cultures. At the end of the surgery, amphotericin 50 mcg/mL was injected subconjunctivally, and gentamycin eye ointment was applied. After that, the antifungal frequency was either maintained or tapered according to the clinical scenario. In follow-up visits, the dimensions of stromal infiltrates and the height of AC hypopyon were documented. Patients were assessed daily until reepithelialization and absence of AC leak were observed. Sutures were removed after 2 weeks.

Elective PK was performed subsequently after complete resolution of infection if the AM filled stroma is obscuring the visual axis. All PKs were done by one surgeon under peribulbar anesthesia with supplementary intravenous (IV) sedation. The donor grafts were 0.5 mm larger than the recipient's corneal flap. Corneal flaps were not removed prior to trephination. Hessburg-Barron trephines were used, and corneal grafts were secured in place with 16 interrupted 10–0 nylon sutures. Postoperatively, patients were prescribed topical ofloxacin 0.3%, prednisolone acetate 1% eye drops, preservative-free lubricant eye drops, and antiglaucoma medications if needed. Topical steroids were tapered gradually over one year. Topography-guided selective suture removal was commenced 3-6 months postoperatively.

Complete resolution of infection and restoration of functioning vision were the study outcome measures.

## 3. Results

Our study included 5 eyes of 5 patients presented with post-LASIK keratomycosis.  Table 1 summarized the demographics and the clinical characteristics of study participants. The mean age of the patients was 22 ± 1.2 (median, 22; range, 19-26) years. The mean duration between LASIK and symptoms onset was 8.8 ± 1 (median, 8; range, 7-12) days, the mean duration from start of symptoms and presentation to us was 14 ± 1.4 (median, 15; range, 10-18) days, and the mean duration between 1^st^ visit and AMT was 12.4 ± 1.2 (median, 12; range, 10-16) days. Steroids were halted by the primary surgeons when infectious keratitis was questioned, and they were prescribed topical antibiotics and antifungal drops. However, case #5 was initially misdiagnosed as diffuse lamellar keratitis (DLK) and treated with steroid wash and increasing the frequency of topical prednisolone acetate 1%.

**Figure 1:**
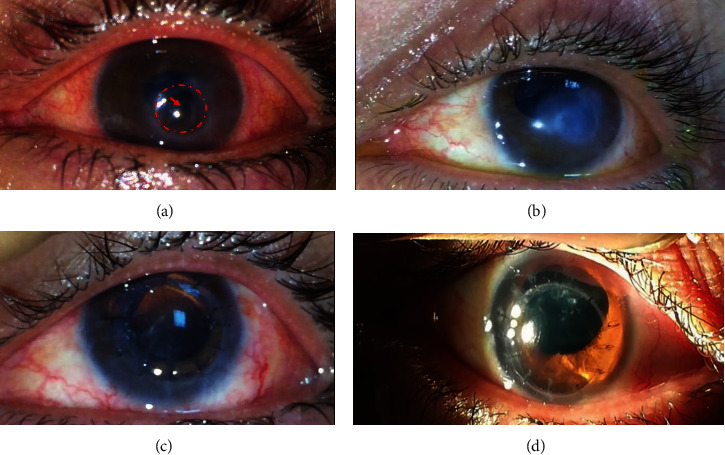
Slit-lamp photos of case #3 showing. (a) Descemetocele and perforation with iris prolapse and inferior AC loss 11 days after intrastromal amphotericin injection. (b) Corneal opacity and epithelialized corneal surface 5 weeks after multilayered fresh amniotic membrane transplantation. (c) Clear full-thickness corneal graft secured with interrupted 10/0 nylon sutures (1^st^ postoperative day). (d) Healthy corneal graft 9 months after PK.

**Figure 2:**
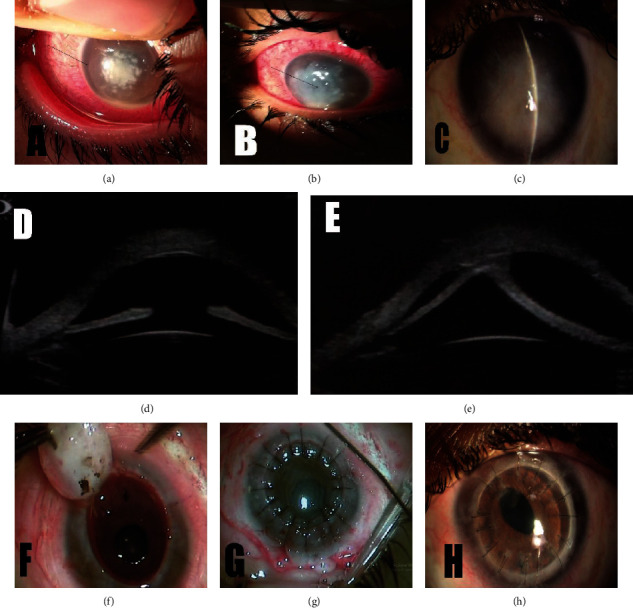
(a) Slit-lamp photo of case #5 at 1^st^ visit showing ciliary injection, central dense interface infiltrates with peripheral satellites within the edge of the flap (arrow) and hypopyon. (b) Slit-lamp photo 48 hours after flap amputation and 2^nd^ intrastromal amphotericin injection showing diffuse dense infiltrate with evolving descemetocele (arrow). (c) Slit-lamp photo, 3 weeks after sutures removal, showing: quiet eye, epithelialized cornea, stroma filled with amniotic membrane, and formed anterior chamber (AC). (d) Ultrabiomicrosopic (UBM) image of anterior segment showing: restored corneal thickness and formed AC. (e) UBM image of anterior segment showing inferior peripheral anterior synechia (PAS). (f, g) Intraoperative pictures captured during penetrating keratoplasty (PK) showing removal of the trephined cornea and release of the PAS at 6 o'clock position (f), corneal graft secured with interrupted 10/0 nylon sutures (g). (h) Slit-lamp photos 5 months after PK showing clear and healthy corneal graft.

The presenting BCVA, clinical features, and antifungal treatment approaches are demonstrated in Table 2. KOH wet mount showed septate fungal hyphae in specimens collected from all patients. Aspergillus fumigatus was detected in all eyes after 72 hours of culture. The LASIK flap was melted and had to be amputated in all eyes except case #3 (Figure 1). Fungal hyphae were seen via microscopic examination of the amputated flap in those cases. Intracameral injection of amphotericin was not done in cases (1, 3, 4) due to high ocular tension. Oral acetazolamide was given to cases (1, 3, 4, and) due to high ocular tension. Case #5 was injected twice intrastromally (5 days apart) due to diffuse dense infiltrates with peripheral satellites (Figure 2). MLF-AMT was performed in all cases after corneal perforation. After AMT, topical amphotericin B 0.15% (every 2 hours) and atropine sulfate 1% (twice daily) were resumed. Specimens collected at time of AMT came back positive for Aspergillus fumigatus. Complete resolution of infection, reepithelialization of corneal surface, and restoration of corneal thickness were seen within 26 ± 1.8 (median, 27; range, 22-32) days after AMT in all cases. Topical treatment was continued for 1 week and tapered over 2 weeks after full resolution of infection. Elective PK was performed uneventfully in all eyes, 2.4 (median, 2; range, 2-3) months after AMT. The final BCVA ranged from 20/20 to 20/80 at an average follow-up of 14 ± 1.1 (median, 14; range, 12-18) months.

## 4. Discussion

LASIK is the most commonly performed refractive surgery for the correction of ametropia. It merges the precision of excimer laser photoablation and the benefits of maintaining the integrity of Bowman's layer and the covering corneal epithelium, decreasing the risk of postprocedure corneal inflammation [12].

Although infection after LASIK, especially fungal infection, is a rare complication, it has serious consequences as visual impairments are not uncommon after infection [13]. Published reports on infections following LASIK found that severe visual acuity reductions were significantly more associated with keratomycosis rather than bacterial and mycobacterial infections [14]. Biomechanically weakening, lack of commercially available antifungal eye drops, usage of steroids [15], and delayed diagnosis might be held responsible for the aggressive course of fungal keratitis after LASIK. It is noteworthy to mention that the high rate of treatment failure in eyes with Aspergillus species may be attributed to the perpendicular growth pattern of fungal filaments that made the infection penetrate quickly deep into the corneal layers [12]. Also, the discrepancy between infection and postoperative sterile keratitis must be made cautiously for vital therapeutic decisions. Misdiagnosis of the infection as inflammation can aggravate the current clinical illness and deteriorate the prognosis (case #5, Figure 2) [3]. Several treatment options have been disclosed in literature such as flap amputation, AMT, and PK along with antifungals. Here, we reported five cases of keratomycosis after LASIK and a stepwise therapeutic plan to reach the optimal outcomes.

Antimicrobial penetration, particularly antifungal drops, may be insufficient to reach infections that lie at the interface or deeper in the stroma owing to the sequestered nature of infections following LASIK [13, 16, 17]. Therefore, it is commended to lift and reposition flap earlier during the course of infection for culture, scraping, and irrigating the stromal bed especially when the infiltrate involves the interface to help better antimicrobial penetration and removes the sequestered nidus for the infection [13, 17]. For case #5, we did intrastromal injections of amphotericin-B (50 *μ*g/ml) twice to increase the ocular concentration of the antifungal therapy enough to be effective in the abolition of the deep corneal infection, and according to Garcia et al., this concentration does not appear to be detrimental to corneal keratocytes or endothelial cells [17]. In addition, we used intracameral injections of amphotericin B at a concentration of 5 *μ*g in 0.1. Yoon et al. postulated that there is no difference in treatment success rates between intracameral amphotericin-B and conventional treatment; however, intracameral injections can decrease time to resolution of hypopyon and time to improvement in the treatment of keratomycosis [18].

Flap amputation was performed in our series except in one case at the initial presentation. Flap amputation may limit the extent of corneal injury caused by the infection and increases drug penetration. Mittal et al. reported a complete resolution of keratomycosis with interface wash using voriconazole with selective flap amputation followed by topical and systemic antifungal treatment [19]. It is noteworthy to mention that failure of the abovementioned treatment has been reported which was attributed to delayed diagnosis [19]. This could be in agreement with our series who failed medical therapy and flap amputation due to relatively late presentation.

AM promotes epithelialization and exhibits antifibrotic, anti-inflammatory, antiangiogenic, and antimicrobial features [20]. There are few reports in the literature that used AM to reestablish globe integrity in cases of perforated keratitis following refractive surgery. AMT stops aqueous humor seepage, seals the corneal defect, and restores AC depth in cases of perforations acting as efficient tectonic support [21, 22]. Also, a ML-AMT using an AM placed in the stromal ulcer provides an alternative to collagen and supplies stromal layers while the overhanging AM graft provides a basement membrane for proper epithelialization [23]. We preferred ML-AM grafts than single layer grafts in managing perforations as a single layer of AM degrades in few weeks which is not a sufficient time for stromal layers to regenerate and fill the defect [24]. In many studies cryopreserved AM was used but in our series we preferred using fresh AMs. Although fresh and preserved AMs have been found to be equally effective when transplanted onto the ocular surface according to Adds et al. [24], Hori et al. demonstrated that viable human amniotic epithelial cells in fresh grafts elicit beneficial effects on the secretion of anti-inflammatory factors, which are otherwise decreased in eyes with infectious keratitis [25, 26]. AMT timing in fungal keratitis is critical, and its application in acute phases of fungal keratitis is controversial. Besides its promoting effect on epithelial healing and mechanical barrier effects, AM may alter the host response against fungi by scavenging the anti-inflammatory cells, including their reactive oxygen species [27]. Therefore, AMT should be considered when the infection is controlled or imminent corneal perforation is suspected [28, 29]. In our series, we used the fresh ML-AMT prior to PK in perforated corneas with active keratomycosis to promote healing and to increase the survival rates of elective corneal transplant. We report 100% resolution of infection and 0% incidence of graft rejection over a mean follow-up of 14 ± 1.4 months.

Compared with visual rehabilitation, the prognosis of PK differs markedly when performed for therapeutic or tectonic reasons. Robaei et al. demonstrated that planned PKs have significantly higher clear graft survival compared with emergency PKs in inflamed tissue [26]. 5-year graft survival rate was 90% for scheduled PKs versus 51% for surgery on keratitis [26]. Maier et al. indicated that TPK that was performed for infectious keratitis exhibited more graft failures than elective keratoplasties [29]. Xie et al. reported a graft rejection rate of 38.5% after TPK in eyes with corneal perforation due to fungal keratitis [30]. Hoffman et al. reported that AMT has decreased the inflammation in severe infectious keratitis and helped to avoid an emergency keratoplasty [31]. They achieved a graft survival rate of 90% over the median 20 months follow-up duration with elective keratoplasty [31], compared to a 2-year graft survival rate of 10.6% after therapeutic keratoplasty performed in fungal keratitis in another study [9]. In our series, after termination of the inflammatory condition of the eye and assessments verified progressive integration of the AM tissues within the cornea, an elective PK was performed to offset residual corneal scarring and improve the visual outcomes. Thus, replacing emergent high-risk keratoplasties by normal risk keratoplasties increase success rate with better visual outcome and no recurrence of infection. Shi et al. reported a rate of 6.34% recurrence in patients with fungal keratitis who underwent keratoplasties [7]. They reported that steroid use before transplantation, corneal perforation, and the presence of hypopyon increases the risk of recurrence significantly. We reported no recurrence in our series, which could be attributed to anterior chamber wash, and intrastromal/subconjunctival injection using amphotericin B that were done during AMT surgery.

Our study has some limitations. First, the lack of serological donor tests 3 months after preparation of fresh AM. Second, all cases in the present study had isolated fungal keratitis, so the effect of fresh AMs was not studied in bacterial or viral keratitis. Third, despite encouraging, our results stem from 5 cases only; however, we hope that other teams replicate our results in their own populations.

In conclusion, fungal keratitis is a vision-threatening complication of LASIK that could be presented with early onset of symptoms in the early postoperative days. Fresh ML-AMT represents a viable method of treatment to promote healing and prevent further melting of corneal tissue induced by fungal keratitis which helps avoid an emergency PK and improves the final visual consequences of a sequential PK in the secondarily quiet eye. Further studies using larger sample sizes and longer follow-up are required to indicate the safety and efficacy.

## Figures and Tables

**Table 1:** Clinical characteristics of patients.

Cases	Age (*Y*)	Gender	Period between LASIK and C/O (D)	Period between onset of C/O and referral (D)	Time to fresh multilayered AMT: indication (D)	Period between AMT and complete resolution of infection (D)	Period between AMT and PK (M), indication of PK	Total follow-up time (M)
1	19	Female	7	10	2.5 mm perforation (14)	22	2, amniotic membrane filled stroma interfering with vision	12
2	22	Male	12	16	3.5 mm perforation (10)	27	2, amniotic membrane filled stroma interfering with vision	14
3	20	Female	10	15	3.5 mm perforation (16)	24	3, amniotic membrane filled stroma interfering with vision	15
4	26	Female	7	12	4 mm perforation (12)	29	2, amniotic membrane filled stroma interfering with vision	12
5	24	Female	8	18	4.5 mm perforation (10)	32	3, amniotic membrane filled stroma interfering with vision	18

**Table 2:** Clinical and microbiological features of patients.

Cases	Medical and surgical interventions	Indication for fresh multilayered AMT	Culture	Initial clinical signs	Presenting BCVA	Prior medical history and surgical interventions	Final BCVA
1	Flap amputation, amphotericin B 50 mcg/ml (interface wash, intrastromal injection), topical (amphotericin B 0.15%, natamycin 5%), oral (itraconazole, doxycycline, acetazolamide)	2.5 mm perforation	Aspergillus fumigatus	Lid edema, chemosis, ciliary injection, cornea (hypothesia, central interface infiltrates 3x3 mm, endothelial plaque, heaped up 3 mm hypopyon, dehiscent LASIK flap), T++	20/200	Topical (moxifloxacin, natamycin, vancomycin, ceftazidime), oral (doxycycline, acetazolamide), LASIK, subconjunctival injection of vancomycin, ceftazidime	20/20
2	Flap amputation, amphotericin B 50 mcg/ml (interface wash, intrastromal and intracameral injection), topical (amphotericin B 0.15%, natamycin 5%), oral (itraconazole, doxycycline)	3.5 mm perforation	Aspergillus fumigatus	Lid edema, chemosis, ciliary injection, cornea (hypothesia, central interface infiltrates 4 × 4 mm, endothelial plaque, heaped up hypopyon, macerated LASIK flap), Tn	CF close to the face	Topical (moxifloxacin, natamycin, vancomycin, ceftazidime), LASIK, subconjunctival injection of vancomycin, ceftazidime	20/40
3	Amphotericin B 50 mcg/ml (interface wash, intrastromal injection), topical (amphotericin B 0.15%, natamycin 5%), oral (itraconazole, doxycycline, acetazolamide)	3.5 mm perforation	Aspergillus fumigatus	Lid edema, chemosis, ciliary injection, cornea (hypothesia, inferior interface infiltrates 4 × 2 mm, endothelial plaque, heaped up 1 mm hypopyon, healthy LASIK flap), T++	HM	Topical (moxifloxacin, natamycin, vancomycin, amikacin), oral (doxycycline), LASIK, subconjunctival injection of vancomycin, amikacin	20/40
4	Flap amputation, amphotericin B 50 mcg/ml (interface wash, intrastromal injection), topical (amphotericin B 0.15%, natamycin 5%), oral (itraconazole, doxycycline)	4 mm perforation	Aspergillus fumigatus	Lid edema, chemosis, ciliary injection, cornea (hypothesia, central interface infiltrates 5 × 4 mm, endothelial plaque, heaped up 1.5 mm hypopyon, macerated LASIK flap), T+	CF close to the face	Topical (moxifloxacin, vancomycin, ceftazidime, natamycin), oral (acetazolamide), LASIK, subconjunctival injection of vancomycin, ceftazidime	20/20
5	Flap amputation, amphotericin B 50 mcg/ml (interface wash, intrastromal and intracameral injection twice), topical (amphotericin B 0.15%, natamycin 5%), oral (itraconazole, doxycycline)	4.5 mm perforation	Aspergillus fumigatus	Lid edema, ciliary injection, cornea (hypothesia, central interface infiltrates 6 × 5.5 mm, endothelial plaque, peripheral satellites, heaped up 3 mm hypopyon, melted LASIK flap), Tn	HM	Topical (moxifloxacin, natamycin, vancomycin, ceftazidime), oral (fluconazole, vitamin C, doxycycline), LASIK, Interface wash with (steroid, moxifloxacin, BSS), subconjunctival injection of vancomycin, ceftazidime	20/80

## Data Availability

The [DATA TYPE] data used to support the findings of this study are included within the article.
